# Supplementary figures and images for: Long-term oncologic outcomes of laparoscopic nephroureterectomy versus open nephroureterectomy for upper tract urothelial carcinoma: a systematic review and meta-analysis
Source: PeerJ. 2016 May 31;4:e2063. doi: 10.7717/peerj.2063 (PMC4893337; doi:10.7717/peerj.2063)

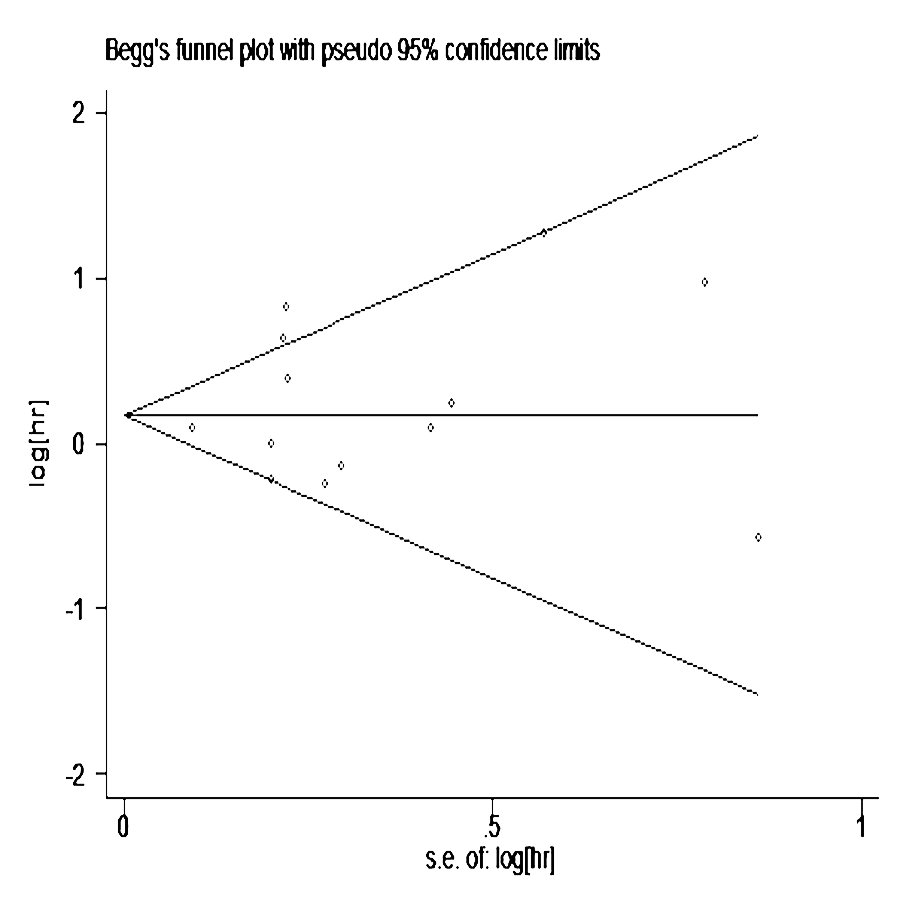

Supplement: Figure S1 [file peerj-04-2063-s001.png]

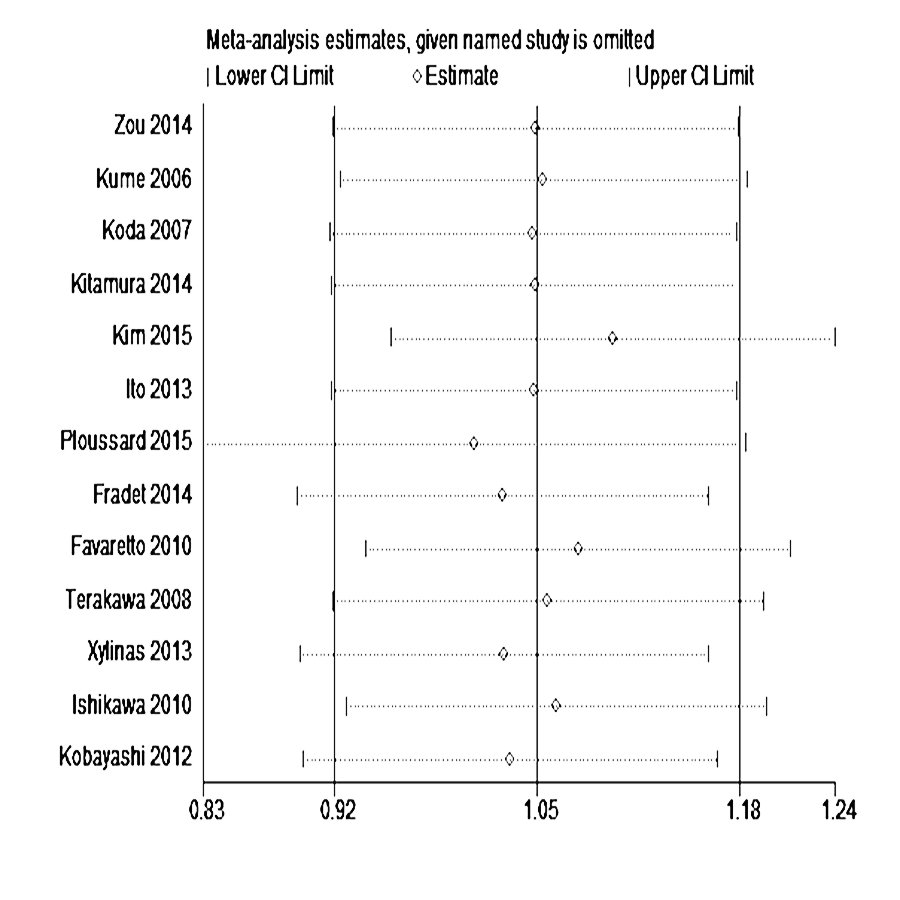

Supplement: Figure S2 [file peerj-04-2063-s002.png]

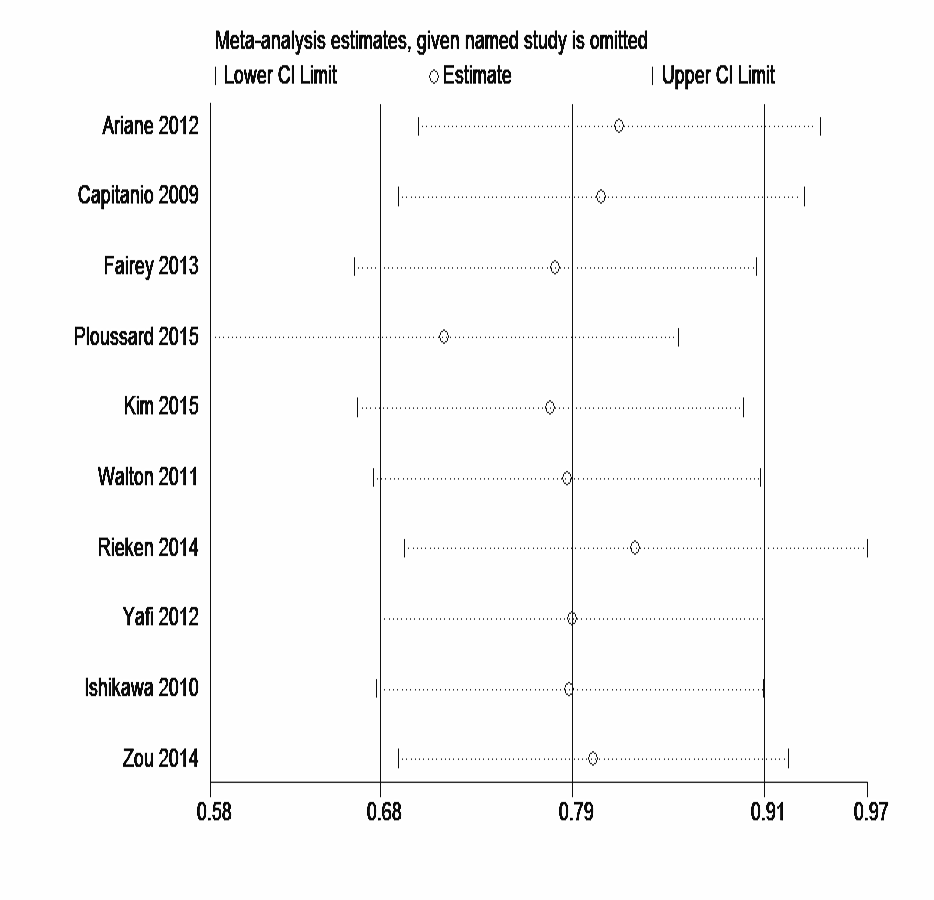

Supplement: Figure S3 [file peerj-04-2063-s003.png]
