# Supplementary material for: Long-term oncologic outcomes of laparoscopic nephroureterectomy versus open nephroureterectomy for upper tract urothelial carcinoma: a systematic review and meta-analysis
Source: PeerJ. 2016 May 31;4:e2063. doi: 10.7717/peerj.2063 (PMC4893337; doi:10.7717/peerj.2063)
Supplement: Supplemental Information 2 [file peerj-04-2063-s005.docx]

**Pubmed**

laparoscop* AND open AND (nephroureterectom* OR ureteronephrectom* OR nephro-ureterectomy) [All Fields]
